# THE RELEASE OF TECHNICAL QUALITY AND PATIENT EXPERIENCE STAR-RATINGS FOR HOME HEALTH AGENCIES AND AGENCY SELECTION

**DOI:** 10.1093/geroni/igz038.2240

**Published:** 2019-11-08

**Authors:** Margot L Schwartz, Momotazur Rahman, Kali S Thomas, R Tamara Konetzka, Vincent Mor

**Affiliations:** 1 Brown University School of Public Health, Providence, Rhode Island, United States; 2 Providence VA Medical Center, Providence, Rhode Island, United States; 3 University of chicago, Chicago, Illinois, United States

## Abstract

To facilitate informed Home Health Agency (HHA) selection for the 3.5 million annual Medicare home health (HH) users, CMS introduced technical quality and patient experience summary star-ratings on the Home Health Compare website in July 2015 and January 2016. There is no information about the relationship between the introduction of these two unique sets of star-ratings and HHA selection. We utilized a conditional logit, discrete choice model, which accounts for all HHAs that each patient could have selected (their “choice-set”) based on ZIP codes, to assess this relationship. We selected a random 5% sample (203,966 admissions) of new Medicare Fee-for-Service HH admissions that occurred in the year before, or the year after star-ratings were released. Star-ratings were obtained from the HH Compare website and categorized as low (=4 stars). We found the introduction of HHA star-ratings was associated with an increased likelihood of selecting an HHA with a high technical quality star-rating, and a decreased likelihood of selecting an HHA with a high patient experience star-rating. After controlling for each patient’s choice-set, patients had 19% increased odds (OR 1.19, 95% CI:1.16,2.23) of selecting a high technical quality HHA and a 12% decreased odds (OR 0.88, 95% CI:0.84,0.92) of selecting a high patient experience HHA, compared to low quality HHAs. Findings suggest patients and referring providers may prioritize technical quality over patient experience. Policy-makers should provide resources to enable HH patients to utilize and interpret the two different HHA star-ratings.

